# Promises and pitfalls of targeted agents in chronic lymphocytic leukemia

**DOI:** 10.20517/cdr.2019.108

**Published:** 2020-05-23

**Authors:** Thomas E. Lew, Mary Ann Anderson, John F. Seymour

**Affiliations:** ^1^Department of Clinical Haematology, The Royal Melbourne Hospital and Peter MacCallum Cancer Centre, Parkville 3050, Australia.; ^2^Blood Cells and Blood Cancer Division, Walter and Eliza Hall Institute of Medical Research, Parkville 3050, Australia.; ^3^Faculty of Medicine, Dentistry and Health Sciences, The University of Melbourne, Parkville 3050, Australia.

**Keywords:** Chronic lymphocytic leukemia, drug resistance, venetoclax, B-cell lymphoma 2, ibrutinib, bruton tyrosine kinase, idelalisib, phosphatidylinositol 3-kinase

## Abstract

Targeted agents have significantly improved outcomes for patients with chronic lymphocytic leukemia, particularly high-risk subgroups for whom chemoimmunotherapy previously offered limited efficacy. Two classes of agent in particular, the Bruton tyrosine kinase inhibitors (e.g., ibrutinib) and the B-cell lymphoma 2 inhibitor, venetoclax, induce high response rates and durable remissions in the relapsed/refractory and frontline settings. However, maturing clinical data have revealed promises and pitfalls for both agents. These drugs induce remissions and disease control in the majority of patients, often in situations where modest efficacy would be expected with traditional chemoimmunotherapy approaches. Unfortunately, in the relapsed and refractory setting, both agents appear to be associated with an inevitable risk of disease relapse and progression. Emerging patterns of resistance are being described for both agents but a common theme appears to be multiple sub-clonal drivers of disease progression. Understanding these mechanisms and developing effective and safe methods to circumvent the emergence of resistance will determine the longer-term utility of these agents to improve patients’ quality and length of life. Rational drug combinations, optimised scheduling and sequencing of therapy will likely hold the key to achieving these important goals.

## Introduction

Chronic lymphocytic leukemia (CLL) is the most common adult leukemia in the Western world, with approximately ~20,100 new cases estimated to be diagnosed in the USA in 2019^[1]^. The chemoimmunotherapy (CIT) era established fludarabine, cyclophosphamide and rituximab (FCR)^[2]^ or bendamustine and rituximab (BR)^[3]^ as the frontline regimens of choice for fit patients, achieving an objective response rate (ORR) of ~90% and complete response rate (CRR) of 30%-44% with durable remissions among many responders. For patients with co-morbidities, frontline therapy with chlorambucil-obinutuzumab achieves an ORR of 76% and CRR 22%, with improved progression free survival (PFS) and overall survival (OS) compared to chlorambucil-rituximab or chlorambucil monotherapy^[4-6]^.

While the development of these effective CIT regimens marked a significant advance in the management of CLL, responses in patients whose disease harboured abnormalities in *TP53* were less frequent and typically short-lived^[7]^, and the toxicity of the most efficacious regimens precluded their use in a high proportion of patients, given the advanced median age and frequent comorbidity at CLL diagnosis^[8]^. The PFS achieved with CIT regimens was also inferior for patients whose disease had unmutated immunoglobulin heavy chain variable region (IGHV) status^[2,9-11]^, reflecting a naïve B-cell originator cell with a more aggressive cancer biology^[12,13]^. These limitations were partially addressed by the advent of targeted agents, most significantly the Bruton tyrosine kinase (BTK) inhibitor ibrutinib^[14]^, the B-cell lymphoma 2 (BCL2) inhibitor venetoclax^[15]^ and the phosphatidylinositol 3-kinase (PI3K) inhibitor idelalisib^[16]^, which maintain response rates within *TP53* aberrant and IGHV unmutated disease and have a favourable toxicity profile compared to CIT. Indeed, emerging data from Phase III trials have demonstrated superior PFS using targeted agent-based therapy over CIT in the frontline treatment of fit^[17]^ and elderly/comorbid patients^[18-21]^, and in the relapsed and refractory (R/R) setting^[22,23]^. The precise therapeutic role and timing of these agents is likely to remain controversial given their cost, restricted geographic approvals and unclear benefit over CIT for fit young patients with IGHV mutated disease^[17,20,24]^, in whom longer-term outcomes after frontline FCR are particularly favourable with > 50% PFS at 12.8 years median follow up^[25]^. Nevertheless, targeted agent-based therapy seems certain to emerge as the most efficacious treatment for most patients with CLL, offering deliverable and effective therapy to those previously unable to reap the benefits of CIT. Despite their advantages, the ultimate utilisation of these therapies in a global context will be restrained by funding limitations^[26]^. Moreover, the longer-term efficacy of novel agents is unfortunately compromised by the relentless development of resistant disease when used in the relapsed and refractory setting^[27,28]^. Further investigation is required to determine the optimal combinations, schedules and sequencing to maximise outcome, as well as the best strategies to prevent or treat emergent resistant disease.

**Figure 1:**
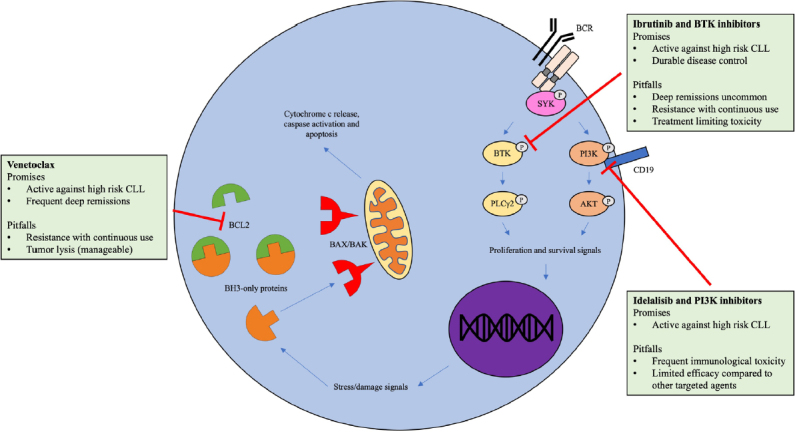
Target agents in chronic lymphocytic leukemia. AKT: v-akt murine thymoma viral oncogene; PI3K: phosphatidylinositol 3-kinase; SYK: spleen tyrosine kinase; BTK: bruton tyrosine kinase; BCR: B-cell receptor; BCL2: B-cell lymphoma 2; CLL: chronic lymphocytic leukemia; BAK: BCL2 homologous antagonist killer; BAX: BCL2 associated X; BH3: BCL2 homology domain 3

In this review, we explore the promises and pitfalls of the three most established targeted agents: venetoclax, BTK inhibitors and PI3K inhibitors [Figure 1]. We outline their mechanism of action, therapeutic potential, drawbacks in clinical use and what is currently known regarding their resistance mechanisms. Richter transformation is a common mechanism of resistance with a unique pathobiology^[29]^, predominantly occurring within 1-2 years of commencement of Phase I/II trials among cohorts enriched for high risk and heavily pre-treated disease^[30,31]^. As this topic has been well reviewed recently elsewhere^[29]^, here we focus on the resistance mechanisms of progressive CLL that foil the promise of targeted therapy.

## Venetoclax

### Mechanism

Venetoclax (ABT-199/GDC-0199) is an orally bioavailable small molecule BCL2 homology domain (BH) 3-only protein mimetic which selectively inhibits the pro-survival protein BCL2^[32]^. BCL2 is constitutively overexpressed in CLL cells and confers resistance to apoptosis by binding to and disabling BCL2 homology domain 3 (BH3)-only proteins, which are instigators of the intrinsic cell death pathway^[33,34]^. When it binds to BCL2 within CLL cells, venetoclax liberates BH3-only proteins from BCL2 sequestration, inducing BCL2 homologous antagonist killer/BCL2 associated X (BAK/BAX) protein mediated mitochondrial permeabilisation and cellular apoptosis^[35]^, independent of p53 function^[36]^. The selectivity of venetoclax for BCL2 was an advance on the therapeutic index of its predecessor, navitoclax (ABT-263), whose demonstrated efficacy in R/R CLL was compromised by dose limiting thrombocytopenia due to on target inhibition of another BCL2 family protein, B-cell lymphoma-extra large (BCL-X_L_), which controls physiologic platelet lifespan^[32,37]^. Consistent with an *in vitro* p53-independent mechanism of cytotoxicity, venetoclax demonstrated a high rate of responses in patients whose disease harboured del(17p), leading to initial US Food and Drug Administration (FDA) approval in 2016 for this high-risk subgroup^[38]^. Based on recently published Phase III trials, venetoclax is now FDA approved for fixed duration therapy in R/R disease in combination with rituximab^[22]^, and as frontline therapy for patients with comorbidities in combination with obinutuzumab^[21]^. Now an established therapeutic option in the treatment of CLL, questions remain regarding the optimal duration and scheduling of venetoclax therapy, appropriate sequencing and combinations with other agents, and the prevention and treatment of resistant disease.

### Promises

The major advantages of venetoclax include its high response rates, even in traditionally high-risk subgroups, and its capacity to induce deep remissions, including undetectable measurable residual disease negative (uMRD) status (< 1 CLL cell per 10^4^ leukocytes by allele-specific oligonucleotide polymerase chain reaction or multiparameter flow cytometry^[39-41]^), with associated prolonged PFS and the option of time limited treatment [Table 1].

**Table 1:** Phase I/II and III trials of venetoclax ± anti-CD20 monoclonal antibodies

Study	Cohort	ORR/CRR MRD	PFS/OS	III/IV toxicity (> 10%)
Phase I/II				
Roberts *et al*.^[15]^ NCT01328626 (venetoclax monotherapy*)	R/R Dose finding (*n* = 56) Expansion (*n* = 60)	79%/20% BM flow cytometry-neg 5%	66% PFS at 15 months 84% OS at 24 months	3 cases clinical TLS, one fatal Neutropenia 41% Anemia 12% Thrombocytopenia 12%
Stilgenbauer *et al*.^[38,42]^ NCT01889186 (venetoclax monotherapy*)	del(17p) R/R (*n* = 158) TN (*n* = 5)	77%/20% PB uMRD 30% BM uMRD 13%	54% PFS at 24 months 73% OS at 24 months	No clinical TLS Neutropenia 40% Anemia 15% Thrombocytopenia 15% Pneumonia 10%
Seymour *et al*.^[43,44]^ NCT01682616 (venetoclax^ - rituximab^+^)	R/R (*n* = 49)	86%/53% BM uMRD 57%	56% PFS at 5 years 89% OS at 5 years	2 cases clinical TLS, one fatal Neutropenia 53% Anemia 14% Thrombocytopenia 16% Febrile neutropenia 12% Infections and infestations 16%
Coutre *et al*.^[45]^ NCT02141282 (venetoclax monotherapy*)	IDEL pre-treated (*n* = 36)	67%/8% PB uMRD 22% BM uMRD 6%	79% PFS at 12 months 94% OS at 12 months	No clinical TLS Neutropenia 50% Thrombocytopenia 25% Anemia 17%
Jones *et al*.^[46]^ NCT02141282 (venetoclax monotherapy*)	IBR pre-treated (*n* = 91)	65%/9% PB uMRD 26% BM uMRD 5%	Median PFS 25 months 92% OS at 12 months	2 cases laboratory TLS Neutropenia 51% Anemia 29% Thrombocytopenia 29% Lymphopenia 1% Febrile neutropenia 11%
Flinn *et al*.^[47]^ NCT01685892 (venetoclax^&^-obinutuzumab^#^)	R/R (*n* = 50) TN (*n* = 32)	R/R 95%/37% PB uMRD 64% BM uMRD 62% TN 100%/78% PB uMRD 91% BM uMRD 78%	R/R 85% PFS at 24 months OS NA TN 91% PFS at 24 months OS NA	Neutropenia 53%-58% Thrombocytopenia 22% Infection 13%-29%
Phase III				
Seymour *et al*.^[22,48,49]^ MURANO NCT02005471 (venetoclax^a^-rituximab^+^ *vs*. bendamustine-rituximab)	R/R VEN + R (*n* = 194)	93%/27% PB uMRD 62% BM uMRD 56%	57% PFS at 4 years 85% OS at 4 years	TLS 3%, 1 case clinical TLS Neutropenia 58% Infection 18%
	BEN + R (*n* = 195)	68%/8% PB uMRD 13% BM uMRD 2%	5% PFS at 4 years 67% OS at 4 years	TLS 1%, 1 case clinical TLS Neutropenia 39% Infection 22%
Fischer *et al*.^[20,21,50]^ CLL14 NCT02242942 (venetoclax^d^-obinutuzumab^#^ *vs*. Chlorambucil-obinutuzumab)	TN CIRS > 6 or CrCl < 70 mL/min VEN + G (*n* = 216)	85%/50% PB uMRD 76% BM uMRD 57%	88% PFS at 24 months 92% OS at 24 months	3 cases lab TLS Neutropenia 53% Infection 18%
	CLB + G (*n* = 216)	71%/23% PB uMRD 35% BM uMRD 17%	64% PFS at 24 months 93% OS at 24 months	5 cases lab TLS Neutropenia 48% Infection 15%

*Continuous therapy until progression, death or other reason to withdraw from trial; ^continuous venetoclax therapy, cessation permitted in good response at clinician discretion; ^&^continuous venetoclax for patients with relapse and refractory disease, fixed duration therapy for 12 months in treatment naïve patients with optional additional 12 months of therapy if not in CR or BM uMRD response; ^+^375 mg/m^2^ in Month 1, then 500 mg/m^2^ each month to a total of six cycles; ^#^100 mg on Day 1, 900 mg on Day 2 (or 1000 mg on Day 1), 1000 mg on Day 8 and 1000 mg on Day 15 of Cycle 1, and subsequently 1000 mg each month to a total of six cycles; ^a^venetoclax continued until progressive CLL, unacceptable toxicity or two years total therapy; ^d^venetoclax continued until progressive CLL, unacceptable toxicity or one year total therapy. ORR: overall response rate; CRR: complete response rate; PFS: progression free survival; OS: overall survival; R/R: relapsed and refractory; TN: treatment naïve; VEN: venetoclax; IDEL: idelalisib; IBR: ibrutinib; BEN: bendamustine; CLB: chlorambucil; R: rituximab; G: obinutuzumab; CIRS: cumulative illness rating scale; CrCl: creatinine clearance; PB uMRD: peripheral blood minimal residual disease less than 10^-4^; BM uMRD: bone marrow minimal residual disease less than 10^-4^; TLS: tumour lysis syndrome; CLL: chronic lymphocytic leukemia

In the first-in-human and subsequently expanded Phase I trial, venetoclax achieved an ORR/CRR of 79%/20% in heavily pre-treated patients (median lines of prior therapy 3, range 1-11). MRD assessment was not protocol-specified and limited in application to patients who achieved a complete remission (CR), confirming bone marrow (BM) uMRD status in 5% of the total population, proving the feasibility of attaining such deep responses in the R/R setting with targeted agent monotherapy. A consistent ORR of 70%-80% was observed in patients whose disease was fludarabine-refractory, bulky (> 5 cm), IGHV unmutated and harbouring del(17p), with CRs observed in all subgroups^[15]^. The Phase II trial enrolling 158 patients with R/R CLL harbouring del(17p) confirmed frequent, deep and durable responses in this subgroup with an ORR/CRR of 77%/20%, peripheral blood (PB) and BM uMRD rate of 30%/13% and a 24-month PFS of 50%^[42]^. Encouraged by evidence of clinical synergy between rituximab and navitoclax^[51,52]^, the Phase Ib venetoclax-rituximab combination study achieved impressive depth of remission in R/R disease, with an ORR/CRR of 86%/53% and BM uMRD status in 61% of patients^[43,44]^. Sixteen patients ceased therapy in uMRD response, with four patients electively withdrawing from the study, 11 patients in ongoing response at a median of 33 (12-58) months off drug and one case of progressive disease at 38 months off therapy^[43]^. This finding, supported by the observation of consistently excellent PFS associated with uMRD remissions regardless of regimen utilised to attain that status from the CIT era^[53-55]^, spurred a new paradigm of time-limited targeted agent combination therapy pursuant of uMRD remission. The MURANO trial compared fixed duration venetoclax therapy for 24 months combined with six months of rituximab to standard CIT with six cycles of BR in patients with R/R CLL, demonstrating significantly higher response rates, uMRD remissions in PB and BM, PFS and OS, with no increase in clinically significant toxicities^[22,48,49]^. Among the 83 patients who attained uMRD remissions, only two developed progressive disease at median of 10 months off therapy and 70% had maintained uMRD status, confirming an enduring benefit for patients who cease therapy in deep response^[49]^. The CLL14 trial in patients with comorbidity or renal impairment combined obinutuzumab with 12 months of either venetoclax or chlorambucil, with increased depth and durability of response in the venetoclax-based treatment arm without additional toxicity, although no significant difference in OS was appreciable with short follow-up at the time of publication^[20,21]^. In both of these Phase III studies, the benefit of venetoclax-based therapy was maintained in both *TP53* mutated and IGHV unmutated disease subsets.

Multiple reports have now confirmed an excellent PFS associated with uMRD attainment with venetoclax-based therapy. Extended follow up of the MURANO and CLL14 trials demonstrated significantly improved PFS for patients who achieved uMRD responses^[48-50]^. Attainment of uMRD status was also associated with prolonged PFS among patients with disease bearing del(17p) treated with venetoclax monotherapy^[42]^. At median survivor follow up of five years, patients achieving uMRD remission with venetoclax monotherapy in Phase I/II trials at our institution had a 92% PFS at two years after attainment of uMRD status^[56]^.

Overall, venetoclax-based therapy outperforms CIT, especially for patients with traditional high-risk disease features. Venetoclax is distinguished from other targeted therapies by its capacity to induce deep remissions with prolonged PFS, rendering it an essential component of time-limited targeted therapy.

### Pitfalls

Although the adverse effects of severe neutropenia and tumour lysis syndrome warrant careful prophylaxis and monitoring, these complications can be prevented or mitigated in the majority of cases. The more troubling pitfall of continuous venetoclax monotherapy, despite its efficacy, is the common emergence of resistant disease with extended follow up, even among many patients who attain uMRD.

Consistent with potent cytotoxicity in CLL, two fatalities due to clinical tumour lysis syndrome (TLS) were reported in the dose ramp-up of the Phase I/Ib studies of venetoclax^[15,44]^. In the Phase I expansion cohort of 60 patients, an extended step-wise dose escalation commencing at 20 mg/day was adopted with a more intensive TLS prophylaxis and surveillance program, with only one case of laboratory TLS and no cases of clinical TLS^[15]^. Davids *et al*.^[57]^ reported a detailed analysis of 166 patients managed with this step-wise dose escalation protocol and risk adapted TLS prevention and management strategy as per manufacturer guidelines^[58]^, identifying five cases of investigator assessed TLS, although none met formal Howard laboratory or clinical criteria^[59]^. Hyperphosphatemia was the most sensitive and earliest indictor of impending TLS. Dose interruptions were applied in four cases, with all patients ultimately resuming therapy and attaining the target dose of 400 mg/day^[57]^. Overall, although clinicians must remain vigilant to the risk of TLS, clinically significant sequelae are rare with the use of established risk adapted protocols. Grade III/IV neutropenia is relatively common, occurring in 37% of patients, predominantly in the first 3-6 months of therapy; however, granulocyte colony stimulating factor treatment and occasional dose interruptions are effective in the majority of cases^[57,60]^. In Phase III trial data, febrile neutropenia was less common with venetoclax-rituximab compared with BR (4% *vs*. 9%), as were aggregated Grade III/IV infections and infestations (18% *vs*. 22%)^[22]^. In CLL14, febrile neutropenia occurred in 5% of patients treated with venetoclax-obinutuzumab compared to 4% with chlorambucil-obinutuzumab, with infections and infestations occurring in 18% and 15%, respectively^[21]^. Although common, venetoclax related neutropenia is typically transient and manageable, and venetoclax-based therapies have comparable infective risk to CIT regimens.

Given the manageable nature of the most common major toxicities, the development of resistant disease with continuous monotherapy emerges as a more challenging threat to patients receiving venetoclax treatment. In the earliest description of progression on venetoclax, fludarabine-refractoriness and complex karyotype (≥ 3 aberrations on conventional metaphase analysis) were the dominant associations with earlier progression in heavily pre-treated, high risk patients receiving venetoclax in Phase I/II clinical trials, although these observations were predominantly driven by early Richter transformation events and may be less relevant to standard risk patients^[30]^. In a pooled retrospective analysis of four Phase I/II trials in patients with R/R CLL, inferior CRR and reduced duration of response was associated with a higher number of prior therapies (especially > 3), prior B-cell receptor (BCR) pathway inhibitor therapy (especially if refractory) and bulky adenopathy^[61]^. As seen in the CIT era, depth of response by international workshop on CLL (iwCLL) criteria and clearance of MRD from the PB were significantly associated with prolonged PFS^[42,56,61]^. Patients whose disease harboured *TP53* or *NOTCH1* abnormalities had an inferior duration of response, despite initial response rates comparable to the overall cohort^[61]^. These mutations are also associated with inferior outcomes with ibrutinib treatment^[62]^. Supportive data from our institutional experience suggest that abnormalities in *TP53* may be associated with an earlier time to recrudescence among patients with uMRD response^[56]^. Although venetoclax induces frequent and deep responses in patients whose disease harbours *TP53* abnormalities, this subgroup appears to nevertheless maintain an increased risk of treatment resistance and disease progression. Overall, the majority of patients with R/R CLL treated with continuous venetoclax monotherapy will ultimately progress at an estimated median of 37 (95%CI: 30-42) months^[61]^. Although patients who attain a deep response have prolonged PFS, MRD recrudescence was common after 3-4-year follow up from attainment of uMRD remission in patients treated with continuous venetoclax monotherapy at our centres^[56]^. Overall, the emergence of resistant disease can be expected for the majority of patients with R/R CLL receiving continuous venetoclax monotherapy.

The mechanisms of venetoclax resistance remain incompletely understood. Primary resistance to venetoclax may be driven by the balance of BH3-only proteins, such as BCL-2-interacting mediator of cell death (BIM), and alternative BCL2 family proteins, such as MCL1 and BCL-X_L_, as observed in *in vitro* data from myeloma cell lines and patients with resistant mantle cell lymphoma^[63]^. Clinical response to navitoclax inversely correlated with MCL1 expression in CLL cells, and was positively associated with higher expression of BIM^[37]^, a BH3-only protein that is liberated by BCL2 inhibition, neutralises MCL1 and directly activates BAX/BAK^[64]^. *In vitro* CD40L and BCR stimulation of CLL cells induces increased expression of BCL-X_L_, Blf-1 and MCL1, with associated resistance to venetoclax, suggesting lymph node microenvironmental signalling may blunt the sensitivity of CLL cells to selective BCL2 inhibition. Preclinical models of microenvironment resistance also suggest that anti-CD20 monoclonal antibodies and BCR pathway inhibitors may deprive CLL cells of these pro-survival signals^[65-67]^, and may explain the higher rates of response and uMRD remission achieved by venetoclax–rituximab and venetoclax-ibrutinib combination regimens compared to historical results with venetoclax monotherapy^[22,44,68,69]^. Although direct head-to-head trials are not available and populations treated in the respective studies differed in a number of important prognostic factors, these combinations appear to enhance response rate and depth, potentially surmounting the primary resistance mechanisms that compromise responses to venetoclax monotherapy.

Insights into secondary (acquired) resistance to venetoclax are likely to mature with extended follow up. The best described mechanism of resistance is acquisition of the Gly101Val point mutation in *BCL2*, which was recently identified by Blombery *et al*.^[28]^ and has been independently confirmed^[70]^. This mutation is not detectable in pre-venetoclax CLL cells or other lymphoproliferative disorders^[28,70]^. The mutation was initially identified in seven out of 15 patients with progressive CLL on continuous venetoclax for R/R disease, including four patients with disease relapsing from uMRD remissions. *in vitro* studies confirmed a 180-fold reduction in venetoclax affinity for the Gly101Val mutated *BCL2*, with resultant resistance to apoptosis^[28]^. The valine substitution disrupts engagement of the P2 binding pocket by venetoclax, while preserving the protein’s capacity to sequester BH3-only proteins^[71]^. Although the Gly101Val mutation is clearly one recurrent mechanism of resistance, the variant allele frequency of the mutation at progression varied significantly (1.4%-70%), suggesting multiple sub-clones with distinct resistance mechanisms. Indeed, an alternative *BCL2* mutation, Asp103Tyr, thought to also disrupt venetoclax engagement with the BCL2 binding site, was identified in addition to the Gly101Val harbouring subclone in one patient^[70]^. This mutation and several other newly identified ones (Asp103Glu, Arg107_Arg110dup and Val156Asp) have also been found to co-occur in 7/10 (70%) patients with progressive disease bearing Gly101Val mutations at our centre, and exceed the variant allele frequency of Gly101Val in several cases. Nevertheless, all patients had significant subpopulations within their progressive CLL which did not harbour any *BCL2* mutations, implying the presence of alternative simultaneous resistance mechanisms^[72]^. In one informative patient, a distinct subpopulation with increased BCL-X_L_ expression was found to be mutually exclusive to the Gly101Val harbouring population, culminating in disproportionately high venetoclax resistance despite a modest Gly101Val allelic frequency. The mechanism of increased BCL-X_L_ expression in these cells is unknown^[28]^. It is likely that numerous mutations and non-point-mutation resistance mechanisms will ultimately be identified in patients with progressive CLL on venetoclax. Another novel resistance mutation in the *BCL2*, Phe104Ile, has been recently described in relapsed follicular lymphoma after venetoclax treatment^[73]^. Amplification of chromosome 1q23, which harbours the loci for MCL1 and PRKAB2 (a regulator of mitochondrial metabolism), has been confirmed in three out of six patients with CLL relapsing on venetoclax. Correlative cell line data support MCL1 upregulation and enhanced mitochondrial oxidative phosphorylation as mechanisms of resistance to venetoclax^[74]^. Whole genome sequencing of eight patients with CLL prior to venetoclax therapy and at time of progression identified recurrent mutations in *BTG1* and homozygous deletion *CDKN2A/B*, as well as one patient each with a mutation in *BRAF* and amplification of PD-L1. *BRAF*^*V600E*^ transduced cell lines exhibited elevated MCL1 expression and *in vitro* venetoclax resistance, offering a potentially druggable target. Phylogenetic tree analysis based on the distribution of mutations indicated heterogenous patterns of clonal evolution which may give rise to concurrent, mechanistically independent resistant clones^[75]^.

Data on the treatment of venetoclax-resistant disease are currently immature. Anderson *et al*.^[30]^ described ten patients with progressive CLL after venetoclax (including four cases after successful salvage of Richter transformation) treated with BTK inhibitor therapy, with objective responses in nine cases, although cohort follow up was short. Other groups have similarly reported responses to ibrutinib in small patient cohorts progressing after venetoclax therapy with limited follow up^[76-80]^. In a recent retrospective analysis of 188 patients receiving subsequent therapy after venetoclax, patients receiving BTK inhibitors had an estimated 24-month PFS of 78%, with longer disease control in BTK inhibitor naïve patients. These outcomes compared to a median PFS of five months with PI3K inhibitors and nine months using chimeric antigen receptor (CAR) T cell therapy^[78]^. Allogenic stem cell transplantation may offer durable disease control for a selected group of patients^[78,81]^. Although ibrutinib appears to be a reasonable treatment for venetoclax-resistant disease, longer-term data are required to establish the ideal therapy for these patients. Re-treatment with venetoclax therapy may be an option after time-limited therapy. In four patients who progressed after ceasing venetoclax-rituximab therapy in good response (two MRD detectable CR and two uMRD CR), re-treatment with venetoclax ± rituximab achieved two partial remissions (PRs) with ongoing response, one PR with progressive disease at 18 months and response status was not evaluable at last follow up for the fourth patient^[43]^. In fourteen patients with progressive disease after completing therapy in the MURANO trial, venetoclax-based retreatment achieved an OR in only two (14%) cases^[48]^, although the efficacy of retreatment may be different for patients whose disease progresses after ceasing therapy in deep remission compared to patients with progressive disease after suboptimal initial response. Extended follow up may clarify whether time-limited, MRD-guided therapy can minimise the selection of resistant clones and facilitate re-treatment with venetoclax-based therapy in some patients.

Taken together, these observations suggest that polyclonal resistance is likely the norm in progressive CLL on continuous venetoclax therapy, synchronously utilising BCL2 mutants, alternative BCL2 family proteins and as yet incompletely defined mechanisms. Open questions remain as to whether combination therapy can eradicate the population from which these resistant clones arise, whether time limited therapy will diminish the selection pressure that drives polyclonal resistance or indeed if these mutations may prove useful biomarkers of impending relapse and the need for treatment intensification. Our current understanding of venetoclax resistance is overwhelmingly derived from patients with R/R disease heavily pre-treated in the chemoimmunotherapy era, and whether these observations will remain relevant in the frontline setting will require further investigation as the experience using venetoclax in treatment naïve (TN) patients matures.

### Summary: venetoclax

We recommend venetoclax for two years combined with six cycles of rituximab for patients with R/R disease, although ibrutinib is a reasonable alternative. For TN patients with comorbidity (cumulative illness rating score > 6) or poor renal function (creatinine clearance < 70 mL/min), we recommend one year of venetoclax combined with six cycles of obinutuzumab, although ibrutinib ± an anti-CD20 monoclonal antibody is also reasonable. Chlorambucil-obinutuzumab is a reasonable treatment for patients with comorbidities if the availability of novel agents is limited. For patients whose disease progresses on venetoclax, we recommend ibrutinib salvage therapy based on limited evidence, although such patients should ideally be enrolled in clinical trials. Among targeted therapies in CLL, venetoclax has the unique capacity to frequently induce uMRD remissions, especially when used in combination. Disease resistance is frequently observed with continuous monotherapy in the R/R setting, driving an argument for time-limited combination therapy pursuant of uMRD remission in current practice and future trials.

## Ibrutinib

### Mechanism

Ibrutinib (PCI-32765) is an orally bioavailable, irreversible small molecule inhibitor of BTK, a member of the Tec kinase family, which is integral to the intracellular communication of BCR stimulation^[82]^. Inhibition of BTK disrupts downstream signalling pathways which enhance CLL cell survival, such as v-akt murine thymoma viral oncogene (AKT), extracellular receptor kinase (ERK) and Nuclear Factor kappa-light-chain-enhancer of activated B cells (NF-kB)^[83-86]^. CLL cells also require intact BTK function to home and adhere to lymphoid organs^[87,88]^, leading to peripheralization of malignant cells with ibrutinib treatment and deprivation of microenvironment survival signalling^[89]^. Extended follow up of Phase I/II studies has confirmed an ORR of 85%-95%, including patients whose disease is heavily pre-treated, bulky, IGHV unmutated or bears del(17p)/*TP53* mutations. Although early CR is uncommon, responses gradually deepen with up to 29% of patients still on therapy at four years ultimately attaining a CR^[14,89-92]^. The strategy of prolonged disease control without deep remission using continuous therapy has been validated in Phase III clinical trials, such that ibrutinib-based therapy has demonstrated superior PFS over CIT in frontline treatment for young^[17]^ and elderly/comorbid patients^[18,19]^, and in the R/R setting^[23]^, although an OS benefit has not been demonstrated in elderly cohorts. Ibrutinib is now FDA approved for the treatment of a broad population of patients with CLL, with and without anti-CD20 monoclonal antibodies; however, the benefit of combination with anti-CD20 therapy is unclear^[19]^. Although ibrutinib is undoubtedly a significant advance in the treatment of CLL, maturing experience with long-term continuous therapy has demonstrated challenges for deliverability^[93]^, and the emergence of resistant disease^[27]^.

### Promises

Ibrutinib-based therapy can achieve long-term disease control for most patients with CLL, including patients with traditional high-risk features and advanced age, and may lead to a degree of immune reconstitution with extended use [Table 2].

**Table 2:** Phase I/II and III trials of ibrutinib ± anti-CD20 monoclonal antibodies

Study	Cohort	ORR/CRR MRD	PFS/OS	III/IV toxicity (> 10%)
Phase I/II				
Byrd *et al*.^[14,89]^ NCT01105247 (ibrutinib monotherapy)	R/R (*n* = 101)	89%/10%	Median PFS 51 months 60% OS at 5 years	HTN 25% Pneumonia 27% Neutropenia 21% Thrombocytopenia 11%
O’Brien *et al*.^[14,92]^ NCT01105247 (ibrutinib monotherapy)	TN **≥** 65 years (*n* = 31)	87%/29%	92% PFS at 5 years 92% OS at 5 years	HTN 32% Pneumonia 10%
Farooqui *et al*.^[90,91]^ NCT01500733 (ibrutinib monotherapy)	Del(17p) TN (*n* = 35) R/R (*n* = 16)	98%/29%	58% PFS at 5 years 76% OS at 5 years	Neutropenia 38% Thrombocytopenia 15%
	Age > 65 TN (*n* = 18) R/R (*n* = 17)	94%/27%	81% PFS at 5 years 84% OS at 5 years	
O’Brien *et al*.^[99]^ RESONATE-17 NCT01744691 (ibrutinib monotherapy)	R/R and del(17p) (*n* = 145)	83%/10%	63% PFS at 24 months 75% OS at 24 months	Neutropenia 18% Pneumonia 13% HTN 13%
Burger *et al*.^[100,101]^ NCT01520519 (ibrutinib-rituximab^$^)	High risk^&^ (*n* = 40)	95%/23% PB uMRD in 2 patients	Median PFS 45 months 73% OS at 36 months	
Jaglowski *et al*.^[102]^ NCT01217749 (Ibrutinib-ofatumumab^#^)	R/R and **≥** 2 prior therapies (*n* = 71)	83%/1.5%	83% PFS at 12 months 89% OS at 12 months	Neutropenia 24% Pneumonia 17%
Abrisqueta *et al*.^[103]^ GELLC7 EudraCT 2016-004937-26 (ibrutinib ± ofatumumab^a^)	TN, CIRS < 6 IBR (*n* = 84) IBR + OFA (*n* = 20)	IBR: 94%/5% +OFA: 100%/40% PB uMRD in 1 patient	98% PFS at 12 months 98% OS at 12 months	Infections 17%
Phase III				
Byrd *et al*.^[23,95,96]^ RESONATE NCT01578707 (ibrutinib monotherapy *vs*. ofatumumab^)	RR IBR (*n* = 195)	91%/11%	Median PFS 44 months 83% OS at 18 months	Neutropenia 20% Pneumonia 10%
	OFA (*n* = 196)	4%/0%	Median PFS 8 months 81% at 12 months	Neutropenia 14%
Burger *et al*.^[97,98]^ RESONATE-2 NCT01722487 (ibrutinib monotherapy *vs*. chlorambucil)	TN **≥** 65 years without del(17p) IBR (*n* = 136)	92%/18%	89% PFS at 24 months 95% OS at 24 moths	Neutropenia 12% Anemia 7% HTN 5%
	CLB (*n* = 133)	35%/2%	Median PFS 15 months 84% OS at 24 months	Neutropenia 18% Anemia 8% Thrombocytopenia 6%
Woyach *et al*.^[19]^ ALLIANCE NCT01886872 (Ibrutinib ± rituximab^+^ *vs*. Bendamustine-rituximab^+^)	TN **≥** 65 years IBR (*n* = 182)	93%/7% BM uMRD 1%	87% PFS at 24 months 90% OS at 24 months	HTN 29% Infection 20% Neutropenia 15% Thrombocytopenia 12% Anemia 12%
	IBR + R (*n* = 183)	94%/12% BM uMRD 4%	88% PFS at 24 months 94% OS at 24 months	HTN 33% Neutropenia 21% Infection 20% Thrombocytopenia 12%
	BEN + R (*n* = 183)	81%/26% BM uMRD 8%	74% PFS at 24 months 95% OS at 24 months	Neutropenia 40% HTN 14% Anemia 12% Infection 15%
Moreno *et al*.^[18]^ iLLUMINATE NCT02264574 (ibrutinib-obinutuzumab^d^ *vs*. Chlorambucil-obinutuzumab^d^)	TN **≥** 65 years or CIRS > 6 or CrCl < 70 mL/min or del(17p) IBR + G (*n* = 113)	91%/41% PB uMRD 30% BM uMRD 20%	79% PFS at 30 months 86% OS at 30 months	Neutropenia 37% Thrombocytopenia 19%
	CLB + G (*n* = 116)	81%/16% PB uMRD 20% BM uMRD 17%	31% PFS at 30 months 85% OS at 30 months	Neutropenia 46% Thrombocytopenia 10%
Shanafelt *et al*.^[17,104]^ NCT02048813 E1912 (ibrutinib-rituximab^+^ *vs*. Fludarabine-cyclophosphamide-rituximab)	TN ≤ 70 years without del(17p) IBR + R (*n* = 354)	96%/17% PB uMRD 8%	89% at 3 years 99% at 3 years	Neutropenia 26% HTN 19%
	FCR (*n* = 175)	81%/30% PB uMRD 59%	73% at 3 years 92% at 3 years	Neutropenia 45% Thrombocytopenia 15% Anemia 15% Infection 10% Febrile neutropenia 16%

Ibrutinib (IBR) used as continuous therapy until progression, death, or other reason to withdraw in all trials. ^&^del(17p) or *TP53* mutated, relapsed del(11q) or relapse within three years of chemoimmunotherapy; ^$^weekly rituximab 375 mg/m^2^ for Week 1-4, then monthly rituximab up to six cycles; ^#^ofatumumab administered as per prescribing manual (300 mg for Dose 1/2000 mg for Doses 2-12) with three groups, namely ibrutinib lead-in, ofatumumab lead-in and simultaneous treatment; ^ofatumumab administered intravenously 300 mg for Dose 1, 2000 mg weekly for seven weeks, then 2000 mg every four weeks up to 24 weeks total; ^+^375 mg/m^2^ in Month 1, then 500 mg/m^2^ each month to a total of six cycles; ^a^seven cycles of ofatumumab added if no CR by Cycle 12 of ibrutinib (300 mg Day 1 and 1000 mg Day 8 of Cycle 13, 1000 mg Day 1 of Cycles 14-18); ^d^100 mg on Day 1, 900 mg on Day 2, 1000 mg on Day 8 and 1000 mg on Day 15 of Cycle 1, and subsequently 1000 mg each month to a total of six cycles. ORR: overall response rate; CRR: complete response rate; PFS: progression free survival; OS: overall survival; R/R: relapsed and refractory; TN: treatment naïve; R: rituximab; CIRS: cumulative illness rating scale; CrCl: creatinine clearance; PB uMRD: peripheral blood measurable residual disease less than 10^-4^; BM uMRD: bone marrow measurable residual disease less than 10^-4^; FCR: fludarabine, cyclophosphamide and rituximab; BEN: bendamustine; CLB: chlorambucil; G: obinutuzumab (100 mg on Day 1, 900 mg on Day 2, 1000 mg on Days 8 and 15, then 1000 mg on Day 1, 28 day cycles)

In a Phase Ib/II study of 51 patients with heavily pre-treated CLL, ibrutinib achieved an initial ORR/CRR of 71%/4%, which increased to 89%/10% on long-term follow up of an expanded cohort (*n* = 101). Despite infrequent CRs, the median PFS for this cohort was 51 months, with 60% OS at five years. Response rates were not compromised in patients with the traditional adverse risk factors of bulky disease, IGHV mutation, complex karyotype or *TP53* abnormalities^[14,89]^. The promising efficacy of ibrutinib in patients whose disease harboured *TP53* abnormalities was validated in the dedicated Phase II study of such patients, with an ORR/CRR of 98%/29% at long-term follow up. Five-year PFS was 74% in TN patients, compared with 19% in patients with R/R disease^[90,91]^. Pooled analysis of 230 patients with R/R CLL with del(17p) treated with ibrutinib had similar results with an ORR/CRR of 85%/10% and 30-month PFS of 57%^[94]^. Overall, these observations compared favourably to frontline FCR for patients with disease bearing del(17p), which achieved a median PFS of 14 months (95%CI: 10-18)^[7]^, and established ibrutinib as a treatment of choice in this high-risk population. Phase II data also suggested promising efficacy in 35 elderly patients (≥ 65 years; 18 TN, 17 R/R), achieving an ORR/CRR of 98%/27% on long-term follow up, with five-year PFS and OS of 81% and 84%, respectively, and no cases of progression or death among TN patients^[90]^. Comparable response rates were observed in cohort of 31 TN patients aged ≥ 65 years, with > 90% five-year PFS and OS^[14,92]^. These data challenged the outcomes previously attained with CIT, and endorsed ibrutinib as a promising option for patients for whom traditional therapy had been undeliverable or ineffective.

Phase III data now support the use of ibrutinib therapy for many of the common clinical scenarios encountered in the management of patients with CLL. Results from the RESONATE trial confirmed the markedly higher efficacy of ibrutinib over ofatumumab in patients with R/R CLL (ORR 91% *vs*. 4%; CRR 11% *vs*. 0%, respectively) and a median PFS of 44 months *vs*. 8 months. This benefit was observed across all subgroups, including disease harbouring del(17p)^[23,95,96]^. In elderly TN patients whose disease lacked del(17p), RESONATE 2 demonstrated the superiority of ibrutinib over chlorambucil in rate and depth of response, PFS and OS, without additional toxicity^[97,98]^. In the iLLUMINATE trial, elderly or comorbid patients were randomised to frontline combination therapy with ibrutinib-obinutuzumab or the previously most effective regimen in this population, chlorambucil-obinutuzumab. Ibrutinib-obinutuzumab showed superior efficacy in terms of ORR (91% *vs*. 81%), CRR (41% *vs*. 16%), PB uMRD attainment (30% *vs*. 20%) and PFS (79% *vs*. 31% at 30 months). The PFS benefit was observed for all subgroups except in patients lacking any of del(17p), *TP53* mutation, del(11q) or unmutated IGHV, although this sub-analysis was small (*n* = 23). No difference in OS has been observed at a median follow up of 31 months^[18]^. The ALLIANCE trial similarly demonstrated superior PFS with ibrutinib-based therapy compared to BR (87% *vs*. 74% PFS at 24 months) in TN elderly patients, with no demonstrated OS benefit (90% *vs*. 95% OS at 24 months)^[19]^.

In younger patients whose disease lacked del(17p), ibrutinib-rituximab demonstrated improved PFS and OS over frontline FCR despite inferior attainment of complete and uMRD remissions, although deepening of response is expected with extended follow up^[17]^. At a median follow up of 45 months, no PFS benefit has been observed in subgroup analysis of patients with IGHV mutated disease^[104]^, in whom FCR can achieve extended remissions^[25]^. The choice of frontline therapy in this subgroup is likely to remain controversial until longer-term follow up data are available. Overall, Phase III data now support the use of ibrutinib-based regimens over CIT for the majority of patients with CLL, with particular benefit for elderly patients not previously eligible for the most effective CIT regimens and patients whose disease bears *TP53* abnormalities in whom durable responses now commonly seen with ibrutinib were previously rare.

The benefit of combining ibrutinib with anti-CD20 monoclonal antibodies is unclear. Although the Phase III trials comparing ibrutinib-based therapy to chlorambucil-obinutuzumab and FCR both utilized concomitant rituximab, the ALLIANCE trial showed no PFS benefit with rituximab combination compared to ibrutinib monotherapy^[19]^. In addition, a recent randomized trial comparing ibrutinib-rituximab to ibrutinib monotherapy for patients with relapsed or *TP53* aberrant CLL found earlier peripheral lymphocyte count normalisation, CR and more frequent attainment of BM uMRD status with combination treatment; however, this did not translate to an improvement in PFS or OS over a median follow up of three years^[105]^. Preclinical data suggest that ibrutinib may impair natural killer cell and macrophage function and thus antagonise the antibody dependent cellular cytotoxicity (ADCC) of rituximab^[106-108]^; it may also downregulate CD20 expression on CLL cells through disruption of microenvironment signals^[109]^. This interference may be largely driven by inhibition of non-BTK kinases such as interleukin-2 inducible tyrosine kinase (ITK), and *in vitro* experiments suggest more selective BTK inhibitors may spare anti-CD20 ADCC^[110]^. Contrary to this hypothesis, acalabrutinib combined with obinutuzumab achieved a low CRR of 8% in a Phase I/II study in R/R CLL^[111]^, compared to a CRR of 3% with acalabrutinib monotherapy^[112]^, questioning the yield of combination. Preliminary data from the Phase III ELEVATE TN study did not demonstrate superior PFS with acalabrutinib-obinutuzumab compared to acalabrutinib alone (30-month PFS 90% *vs*. 82%), although CRs appear more frequent (13% *vs*. < 1%) and longer-term follow up is required^[113]^. The ostensibly increased speed and depth of response observed with ibrutinib-obinutuzumab in the iLLUMINATE trial suggests an enhanced clearance of malignant cells with combination therapy^[18]^, and the enhanced cytotoxicity of obinutuzumab may be less compromised by ibrutinib co-administration than rituximab^[114-118]^. In two similarly designed Phase II trials combining ibrutinib with obinutuzumab or ofatumumab after bendamustine debulking treatment, patients in the obinutuzumab trial achieved a higher rate of PB uMRD response compared to the ofatumumab group (48% *vs*. 14%), further endorsing the possibility that obinutuzumab may be the superior partner to ibrutinib over earlier generation anti-CD20 monoclonals^[119,120]^. Ultimately, longer-term follow up of randomized studies will be required to establish the role of anti-CD20 monoclonal combination, which may be influenced by the kinome of the BTK inhibitor or the properties of the monoclonal antibody. Whether the improved PFS and OS over FCR observed in younger, TN patients in the E1912 trial is dependent on concomitant rituximab is unclear, as is the benefit of ibrutinib-obinutuzumab combination over ibrutinib monotherapy in elderly and comorbid patients.

Immune reconstitution may also represent an advantage of ibrutinib therapy. Sun *et al*.^[121]^ observed increased serum IgA levels in patients with CLL receiving ibrutinib, with fewer infections in patients with greater IgA improvements. Ibrutinib can increase CD4+ and CD8+ numbers, reduce CLL mediated immunosuppression^[122]^ and may enhance Th1 subtype activity and anti-parasite immunity through ITK inhibition^[123]^. Preliminary data also suggest ibrutinib may enhance the proliferative capacity and efficacy of CAR T cells, possibly through reduced expression of programmed death protein 1 (PD1) on T cells^[124-126]^, and expand host CD8+ subpopulations with cytotoxicity against CLL cells^[127]^. In contrast, however, ibrutinib has also been shown to compromise BTK-dependent macrophage activation in response to *Aspergillus fumigatus* infection in mouse models^[128]^. Initial case reports suggested an association between ibrutinib therapy and invasive fungal infections^[129-132]^, including a high rate of invasive *Aspergillosis* in patients receiving the agent for central nervous system lymphoma^[133,134]^. A retrospective review of 33 invasive fungal infections in patients receiving ibrutinib at French centres identified *Aspergillus* species as the most common organisms (27/33), with cerebral involvement in 40% of cases^[135]^. In an analysis of 459 patients receiving ibrutinib for lymphoma at the Memorial Sloan Kettering Cancer Centre, 16 (4.2%) instances of invasive fungal infections were identified, with proven or probable invasive aspergillosis accounting for 50% of cases. The remainder were *Pneumocystis jiroveci* pneumonia (PJP) (*n* = 4), pulmonary *Cryptococcus* (*n* = 3) and *C. albicans* fungemia (*n* = 1)^[136]^. In another review of 566 patients receiving ibrutinib for lymphoma, opportunistic infections occurred in 2.3% of patients at six months and 4.7% of patients at five years, predominantly due to fungal (mainly *Aspergillus*) infections with no cases of PJP. Opportunistic infections were not observed in patients receiving ibrutinib as frontline therapy and were associated with higher number of previous treatments, suggesting that the cumulative immunosuppressive effects of prior therapies may contribute to the risk^[137]^. Overall, opportunistic infections appear to be an uncommon but serious adverse effect of ibrutinib therapy. The immune effects of ibrutinib are clearly multifaceted and mediated by on target and off target kinase inhibition. The immunological impact of BTK inhibitor therapy warrants further study, particularly in more selective BTK inhibitors for which the longer-term immunological effects are unknown^[138,139]^.

### Pitfalls

Although ibrutinib achieves improved disease control for the majority of patients, the rare attainment of deep responses (especially uMRD) demands continuous use, leading to several emerging concerns. Discontinuation of therapy due to cumulative toxicity is common, especially in the elderly and patients outside of clinical trials. Despite significant activity against disease with del(17p)/*TP53* mutations and complex karyotype, extended follow up has identified inferior PFS in these patients in the longer term. For patients who are able to tolerate long-term therapy, the emergence of resistant disease is common if not inevitable, commonly bearing *BTK* point mutations at Cys481 that disable irreversible inhibition by ibrutinib.

With continuous use, treatment limiting toxicities with ibrutinib are commonly encountered. In a pooled analysis of 616 patients treated with ibrutinib in clinical trials and routine practice, 41% of patients discontinued therapy at a median of seven months. Toxicity was the most common reason for cessation, attributable to 63% of treatment terminations in TN patients and 50% of patients with R/R disease. By comparison, progressive disease was a less common reason for cessation, accounting for 16% of TN and 21% of previously treated patients^[93]^. Similar data have been observed in another recent description of 205 patients receiving ibrutinib outside of clinical trials (42% discontinuation due to adverse effects and median time to discontinuation of nine months)^[140]^. Among young fit TN patients treated with ibrutinib-rituximab in the E1912 trial, the rate of cessation due to adverse effects or complications was lower (14% at a median follow up of 45 months), but was nevertheless the most common reason for cessation, accounting for 51% of terminations (compared to 24% due to progressive disease). Higher cumulative illness rating scale scores were associated with a higher rate of treatment cessation for reasons other than progressive disease or death^[104]^. These observations indicate that the potential durable disease control offered by ibrutinib therapy is often compromised by treatment limiting toxicity. The most common adverse effects accounting for treatment termination were arthralgia, atrial fibrillation, rash, infection, bleeding and diarrhea^[93]^, with older, heavily pre-treated and comorbid patients more likely to discontinue due to toxicity^[31,104,141]^. In the TN setting, 41% of toxicity-driven cessation was due to arthralgia, but published data on the pathophysiology and management of this phenomenon are lacking^[93]^. Bleeding affects up to half of all patients treated with ibrutinib, although this is typically minor, with a cumulative major haemorrhage rate of 9% at five-year follow up^[14]^. The ibrutinib bleeding diathesis is likely mediated through on target and off target kinase inhibition, supported by *in vitro* data and the observation that congenital BTK deficiency in X-linked agammaglobulinemia is not associated with increased bleeding^[142-144]^. Atrial fibrillation occurs in approximately 10% of patients over 36 months^[145]^, and may be mediated by decreased PI3K-AKT signalling in cardiomyocytes due to BTK and Tec protein kinase inhibition^[146]^. More concerningly, ibrutinib appears to increase the risk of sudden cardiac death and ventricular arrhythmias. Pooled analysis of patients receiving ibrutinib have estimated an incidence of 617-788 events per 100,000 person-years, compared to the expected rate among healthy 65 year olds of 200-400 events per 100,000 person-years^[147,148]^. Seven instances of unexplained sudden death (4%) occurred in the ibrutinib monotherapy arm of the ALLIANCE trial compared to two cases in the BR cohort (1%), potentially contributing to the failure to recapitulate the survival benefit over CIT seen in younger patients^[19]^. Similarly, seven cases of ventricular arrhythmia or sudden death occurred in the ibrutinib arm of the HELIOS trial (2%) compared to no events in the placebo arm^[149]^. Estimates for the median time to first ventricular event from ibrutinib initiation vary from 2 to 16 months, with cases reported as early as five days on therapy and beyond four years^[147,148]^. Although these events are rare, the phenomenon is concerning, especially considering the increasing options and life expectancy for patients with CLL. Overall, toxicity significantly compromises the deliverability of continuous ibrutinib therapy, calling for the development of less toxic, more selective BTK inhibitors or the use of combination therapy to achieve deep remission that facilitates drug cessation.

Acalabrutinib (ACP-196) is a second-generation BTK inhibitor without irreversible inhibitory action against epidermal growth factor receptor, TEC and ITK, as seen with ibrutinib. In initial data from a Phase I/II study in patients with R/R CLL, acalabrutinib maintained a high ORR of 95% with no atrial fibrillation after 14-month follow up, although arthralgia and petechial bleeding both occurred in 16% of patients^[138]^, and one Grade III bleeding event (epistaxis) occurred with extended follow up^[112]^. Similarly high ORR has been observed TN patients, with Grade III/IV atrial fibrillation, bleeding and hypertension occurring in 2%, 3% and 7% of patients, respectively, at median time on study of 42 months^[150,151]^. Among 60 patients intolerant of ibrutinib with progressive CLL treated with acalabrutinib, 67% of patients remained on drug at a median follow up of 19 months, with 13% ceasing due to progression and 10% due to adverse effects. Atrial fibrillation and major haemorrhage occurred in three and two patients, respectively^[152]^. Overall, these preliminary data suggest that acalabrutinib is active against CLL, and may reduce, but not preclude, the toxicities seen with ibrutinib.

Tirabrutinib (ONO/GS-4059), a similarly selective BTK inhibitor, achieved objective responses in 96% of patients with R/R CLL in a Phase I clinical trial, with no atrial fibrillation observed after a median follow up of three years, although one Grade III haematoma has been reported and low grade arthralgias and bruising are common^[153,154]^. A third selective BTK inhibitor, zanubrutinib (BGB-3111), has also shown preserved efficacy in CLL (ORR/CRR 97%/14%), with infrequent major bleeding and atrial fibrillation (2%-3% of patients after a median follow up of 27 months) and more patients ceasing due to progressive disease than adverse effects (11% *vs*. 3%)^[139,155]^. Together with high early response rates, the relative rarity of major bleeding and arrhythmias with these agents is particularly encouraging, and support the currently active trials which interrogate whether selective BTK inhibition can preserve therapeutic efficacy while minimising toxicity.

Another potential strategy to manage ibrutinib adverse effects is dose reduction. Several retrospective analyses of patients treated outside of clinical trials found no evidence of inferior disease control among patients who required dose reductions^[156-158]^. Phase I data suggest that a progressive reduction from 420 mg/day in Cycle 1 to 140 mg/day by Cycle 3 does not reduce biochemical BTK occupancy^[159]^. Although prospective clinical data are required to determine the impact of dose reduction on disease control and adverse effects, current data suggest that a trial of dose reduction in response to adverse effects may not necessarily compromise treatment efficacy.

Unfortunately, for patients who are able to tolerate long-term ibrutinib, the development of resistant disease is common, if not inevitable, among patients with previously treated or *TP53* aberrant disease^[14,90,96]^. Primary resistance to ibrutinib is unusual in CLL, although extensive prior treatment and bulky disease are associated with inferior CR attainment on multivariate analysis^[160]^. In a cohort of 70 patients treated with ibrutinib, higher levels of surface IgM were associated with earlier time to progression on univariate analysis, possibly due to IgM mediated preservation ERK signalling despite BTK inhibition^[161]^. In a retrospective pooled analysis for four Phase II and III trials, the pre-treatment risk factors of *TP53* mutations, advanced Rai stage, elevated beta-2-microglobulin and prior treatment were associated earlier development of resistant disease^[162]^. At five-year follow up of early phase trials, del(17p) and greater number of prior therapies were associated with inferior PFS and OS on multivariate analysis^[14]^. Concordantly, extended follow up of the RESONATE trial suggested that patients whose disease harboured del(17p) and/or *TP53* mutations had an inferior PFS compared to patients whose disease had neither abnormality^[95,96]^. In a retrospective analysis of 616 patients, predominantly with R/R disease, complex karyotype, but not del(17p), was associated with inferior PFS^[93]^, supporting similar observations that complex karyotype is the more potent adverse prognostic indicator^[94,141,163,164]^. Overall, these data suggest that patients whose disease contains *TP53* abnormalities have an inferior duration of response to ibrutinib, although this may be driven predominantly by the enrichment for complex karyotype within this group. Similar to observations in venetoclax-treated patients, *TP53* abnormalities appear to facilitate acquisition of secondary resistance mechanisms and subsequent progressive disease without compromising initial responses to targeted therapy.

The most well described mechanisms of secondary resistance are acquisition of mutations in the *BTK* gene at the Cys481 residue or, less commonly, activating mutations in phospholipase C gamma 2 (*PLCG2*). These mutations were first described in six patients with progressive CLL, all of whom had del(11q), del(17p) or complex karyotype at ibrutinib commencement. The Cys481Ser mutation at the ibrutinib binding site degrades BTK inhibition from irreversible to reversible, restoring downstream ERK and AKT signalling. The gain-of-function *PLCG2* mutations enable BTK-independent signalling downstream of the BCR, similarly reactivating ERK and AKT activity. Many mutations lie within the Src homology domain 2 (SH2) autoregulatory domain of *PLCG2*, accounting for the constitutive phosphorylation and activation conferred^[27]^; however, the mechanism of other described mutations outside the SH2 domain is less clear^[165]^. Variant substitutions at the Cys481 residue, alternative *BTK* mutations and diverse *PLCG2* mutations have now been identified^[31,166-170]^, altogether occurring in 85% of patients relapsing on ibrutinib in the largest published dataset^[141]^, comparable to other groups^[169,171]^. *BTK* mutations also appear to be common among patients relapsing on acalabrutinib^[169,172]^. An alternative *BTK* mutation Leu528Trp has recently been shown to co-occur with Cys481 mutations in patients whose disease progresses on zanubrutinib, although this mutation appears to compromise the enzymatic activity of BTK^[173]^. As seen with point mutations conferring resistance to venetoclax, these mutations have not been identified in pre-treatment samples^[174]^. Mutations have been detected up to 18 months prior to clinical progression, with a median time from detection to clinical relapse of nine months^[141]^. Although these mutations are clearly relevant to resistance, 15%-20% of patients progressing on ibrutinib likely harbour an alternative mechanism. Furthermore, variant allele frequency of *BTK* mutations are low (< 10%) in some disease at progression, which may be due compartmental nodal relapse or, as suspected with venetoclax resistance, polyclonal progression utilizing multiple resistance mechanisms^[141]^. Comparison of gene sequencing results pre- and post-BTK inhibitor therapy identified new mutations in *TP53, SF3B1, NOTCH1, POT1* and *CARD11* in seven patients who progressed on ibrutinib without *BTK/PLCG2* mutations^[169]^. A recent analysis of 180 patients treated with single agent ibrutinib identified an association between *NOTCH1* mutations and inferior nodal response, PFS and OS, supporting its putative role in ibrutinib resistance^[62]^. Infrequent co-acquisition of these new mutations with *BTK/PLCG2* mutations indicates potentially independent mechanisms. Moreover, disease harbouring *BTK/PLGC2* mutations had a significantly longer time to clinical progression compared to alternative mutants, which may imply a proliferative disadvantage in the *BTK/PLCG2* mutated clone^[169]^. In patients without these mutations, expansion of sub-clones harbouring del(8p) has been reported as an alternative mechanism of resistance, potentially through decreased expression of the tumour necrosis factor alpha apoptosis-inducing ligand (TRAIL) receptor and failure of TRAIL-induced apoptosis^[167]^.

Potential therapeutic strategies for *BTK/PLCG2* mutated CLL include inhibition of BCL2, disruption of alternate BCR pathway kinases and the use of novel BTK inhibitors which do not rely on the Cys481 residue for binding. In patients with progressive CLL after ibrutinib treatment, venetoclax achieved an ORR 65%, CRR of 9% and PB uMRD status in 26%, with an associated median PFS of 25 months. Among 17 patients with *BTK/PLCG2* mutations, venetoclax achieved a response in 12 (71%) patients (11 PR; 1 CR), with a falling *BTK* Cys481Ser allelic frequency in all cases assessed and no significant difference in PFS between patients with and without mutations^[46]^. Suppression of the *BTK/PLCG2* variant allelic burden by venetoclax has also been reported by another group^[169]^, arguing that such clones retain BCL2 dependence. In a retrospective analysis, idelalisib achieved an ORR of 46% (CRR 0%) and a median PFS of nine months in patients who discontinued ibrutinib^[175]^, suggesting venetoclax may be the more effective agent in this setting. Preliminary data from the Phase II COSMOS trial demonstrated frequent responses in heavily pre-treated patients whose disease had progressed after BTK inhibitor therapy using the anti-CD19 monoclonal antibody tafasitamab in combination with idelalisib or venetoclax, achieving PB uMRD status in 1/11 (9%) and 6/13 (46%) patients, respectively^[176]^. Duvelisib, an inhibitor of PI3K isoforms d and g, demonstrated poor response rates in R/R CLL previously treated with ibrutinib in a Phase I study^[177]^. Despite the biological rationale of inhibiting kinases upstream of BTK and PLCG2, the spleen tyrosine kinase (SYK) inhibitor entospletinib (GS-9973) achieved an ORR of only 24% and a median PFS of 3-8 months in ibrutinib exposed R/R CLL^[181]^. Vecabrutinib (SNS-062), a reversible BTK inhibitor independent of the Cys481 residue, has *in vitro* cytotoxicity against *BTK* Cys481-mutated cell lines^[179,180]^ and preliminary safety data in patients with CLL^[173]^. Similarly, LOXO-305 is an oral bioavailable selective non-covalent BTK inhibitor that can interrupt Cys481 mutated BTK, with responses in all CLL patients treated in a Phase I study, including one patient with disease harbouring the Cys481Ser mutation^[182-184]^. Several other BTK inhibitors are in development and may ultimately prove efficacious against *BTK* mutated disease^[185-189]^. Currently, venetoclax appears to be the best supported therapy for patients progressing with CLL on ibrutinib. Given that *BTK* and *PLCG2* mutations are detected after a median of nine months on ibrutinib monotherapy^[141]^, it remains unknown if time-limited venetoclax-ibrutinib combination will restrain their emergence, or obviate the continuous ibrutinib exposure that drives their selection.

### Summary: ibrutinib

Phase III trial data endorse ibrutinib as an effective and durable treatment for most of the common clinical scenarios within CLL; however, deep remissions are rare and continuous therapy is required. We recommend ibrutinib-based therapy as a frontline for most patients, especially those with *TP53* abnormalities. Fit, young patients with *TP53* wildtype, IGHV mutated disease should be counselled on the merits of FCR or ibrutinib-rituximab therapy. For patients with established cardiovascular comorbidity, we favour venetoclax-based therapy if available. Chlorambucil-obinutuzumab is a reasonable treatment for patients with comorbidities if the availability of novel agents is limited. We recommend venetoclax-rituximab for the treatment of ibrutinib resistant CLL. Inevitable resistance and treatment-limiting toxicity currently complicate continuous ibrutinib therapy, and we hope these will be addressed by longer-term follow up of more targeted BTK inhibitors and time-limited novel agent combination therapy.

## Idelalisib

### Mechanism

Idelalisib (GS-1101/CAL-101) is a selective inhibitor of the p110d isoform of PI3K, an enzyme downstream of the BCR responsible for transduction of pro-survival signals in CLL^[190]^. The d isoform of PI3K is highly expressed in lymphoid cells, and inhibition of this enzyme interrupts downstream AKT signalling^[190,191]^ and microenvironment protection from apoptosis^[66,192]^. Based on Phase III trial data, idelalisib is currently FDA approved for treatment of R/R CLL in combination with rituximab^[16]^.

### Promises

Despite only modest efficacy in the Phase I/II monotherapy trials^[191,193]^, idelalisib combined with rituximab or ofatumumab achieved an ORR/CRR of 83%/5% in patients with R/R CLL, with a median PFS of 24 months^[194,195]^. In a Phase III trial comparing rituximab with and without idelalisib for patients with R/R CLL unable to receive CIT, idelalisib-rituximab combination demonstrated an ORR of 81% (no CRs) and superior PFS and OS (66% *vs*. 13% PFS at 12 months; 92% OS *vs*. 80% OS at 12 months). As seen in other targeted therapies, the PFS benefit was maintained in patients whose disease harboured del(17p)/*TP53* mutations or was IGHV unmutated^[16]^. The published clinical trial data for idelalisib in CLL are summarised in Table 3.

**Table 3:** Phase I/II and III trials of idelalisib ± anti-CD20 monoclonal antibodies

Study	Cohort	ORR/CRR MRD	PFS/OS	III/IV toxicity (> 10%)
Phase I/II				
Brown *et al*.^[191]^ NCT00710528 NCT01090414 (idelalisib monotherapy)	R/R CLL (*n* = 54)	72%/0%	Median PFS 16 months 75% OS at 3 years	Neutropenia 43% Pneumonia 20% Neutropenic fever 11%
Furman *et al*.^[194,195]^ (idelalisib + rituximab^%^ or ofatumumab^)	R/R CLL (*n* = 40)	83%/5%	Median PFS 24 months 80% OS at 24 months	Diarrhea/colitis 23% Pneumonia 18%
O’Brien *et al*.^[200]^ NCT01203930 (idelalisib-rituximab^%^)	TN ≥ 65 years (*n* = 64)	97%/19%	PFS 83% at 3 years OS 90% at 3 years	Diarrhea/colitis 42% Neutropenia 28% ALT/AST elevation 23% Pneumonia 19%
Zelenetz *et al*.^[193]^ (idelalisib monotherapy)	TN ≥ 65 years (*n* = 37)	81%/0%	NA	Neutropenia 20%
Lampson *et al*.^[201]^ NCT02135133 (idelalisib-ofatumumab^)	TN (*n* = 27)	89%/4%	Median PFS 23 months OS 88% at 3 years	ALT/AST elevation 52% Neutropenia 33% Colitis/diarrhea 15%
Phase III				
Coutre *et al*.^[202]^ NCT01088048 (idelalisib ± rituximab^%^ ± bendamustine)	R/R CLL IDEL + R (*n* = 19)	90%/0%	Median PFS 37 months	Neutropenia 26% Pneumonia 21% Diarrhea 16% Febrile neutropenia 16%
	IDEL + BEN (*n* = 18)	78%/6%	Median PFS 19 months	Neutropenia 67% Thrombocytopenia 22% Anemia 28% AST/ALT elevation 22% Pneumonia 22% Sepsis 22% Febrile neutropenia 17%
	IDEL + BEN + R (*n* = 15)	88%/13%	Median PFS 23 months	Neutropenia 60% Anemia 13% Diarrhea 13% Pneumonia 13% Rash 13%
Furman *et al*.^[16,203]^ NCT01539512 (idelalisib-rituximab^#^ *vs*. rituximab monotherapy^#^)	R/R CLL with: myelosuppression, CrCl < 60 mL/min or CIRS > 6 IDEL + R (*n* = 110)	81%/0%	66% PFS at 12 months 92% OS at 12 months	Neutropenia 34% Thrombocytopenia 10%
	R (*n* = 110)	13%/0%	13% PFS at 12 months 80% OS at 12 months	Neutropenia 22% Thrombocytopenia 16% Anemia 14%
Jones *et al*.^[204,205]^ NCT01659021 (idelalisib-ofatumumab^ *vs*. ofatumumab monotherapy*)	R/R CLL IDEL + OFA (*n* = 174)	75%/0%	Median PFS 16 months Median OS NR	Neutropenia 34% Diarrhea 23% Pneumonia 20%
	OFA (*n* = 87)	18%/0%	Median PFS 8 months Median OS NR	Neutropenia 16%

Idelalisib (IDEL) used as continuous therapy until progression, death, or other reason to withdraw in all trials. ^%^375 mg/m^2^ weekly for 6-8 weeks; ^#^375 mg/m^2^ for first dose, 500 mg/m^2^ every two weeks for four doses, then 500 mg/m^2^ every four weeks for three doses (total eight doses). OFA^: ofatumumab intravenously 300 mg Week 1, 1000 mg Weeks 2-7, 1000 mg Q4 weekly × 4; OFA*: ofatumumab intravenously 300 mg Week 1, 2000 mg Weeks 2-7, 2000 mg Q4 weekly × 4. ORR: overall response rate; CRR: complete response rate; PFS: progression free survival; OS: overall survival; R/R: relapsed and refractory; TN: treatment naïve; R: rituximab; BEN: bendamustine; CIRS: cumulative illness rating scale; CrCl: creatinine clearance; PB uMRD: peripheral blood measurable residual disease less than 10^-4^; BM uMRD: bone marrow measurable residual disease less than 10^-4^

### Pitfalls

Although idelalisib represents another targeted therapy with efficacy in traditional high-risk disease, the depth and duration of response seen in Phase III clinic trials is modest compared with outcomes in comparable trials with ibrutinib and venetoclax^[23,44,95,96]^. In a retrospective analysis, patients receiving ibrutinib as first BCR pathway inhibitor in either the frontline or R/R setting had superior PFS compared to patients receiving idelalisib as initial therapy^[175]^. In the ASCEND trial, patients with R/R CLL were randomised to receive acalabrutinib monotherapy or investigator’s choice of idelalisib-rituximab or bendamustine-rituximab, with the majority of investigators selecting idelalisib-rituximab (199 patients *vs*. 36 patients bendamustine-rituximab). Acalabrutinib treatment was associated with a significantly prolonged PFS (12-month PFS 88% *vs*. 68%) and superior tolerability compared to investigator’s choice^[196]^, strengthening the argument for BTK inhibitors over PI3K inhibitors. Compounding this apparently inferior efficacy are concerns regarding deliverability, with 94% of patients discontinuing therapy at a median of six months in a large retrospective analysis, more commonly due to toxicity than progressive disease (45% *vs*. 28% of discontinuations, respectively). Immune phenomena, particularly pneumonitis, colitis, rash and transaminitis, were the most common reasons for cessation^[175]^. Although poorly understood, these events may be related to inhibition of PI3K p100d in regulatory T cells, leading to enhanced CD8+ T cell activity^[197,198]^ and Th17 phenotype^[199]^. In one young TN cohort treated with idelalisib and ofatumumab, Grade ≥ III immune mediated hepatotoxicity occurred in 52% of patients, and 55% of patients discontinued therapy at a median of eight months due to toxicity^[201,206]^. In contrast, a pooled analysis of 760 patients observed Grade ≥ III transaminitis in only 13%-16% of subjects^[207]^, suggesting these adverse effects may be more common in younger patients treated in the frontline. Two deaths due to immune mediated colitis and hepatitis led to termination of a Phase I trial of lenalidomide, idelalisib and rituximab^[208]^, and frequent severe pneumonitis prohibited the combination of idelalisib with entospletinib^[209]^. Given these observations regarding modest efficacy and treatment limiting toxicity, durable disease control with idelalisib is less common than with ibrutinib or venetoclax. In contrast to venetoclax and ibrutinib, Phase III trials supporting idelalisib over other established treatments have not emerged, confining its use to the R/R setting.

Although progressive disease on idelalisib does occur, the mechanisms for resistance are poorly understood. Point mutations at the active binding site, as seen in ibrutinib and venetoclax resistance, have not been identified on whole exome sequencing of patients relapsing on idelalisib^[210]^. In a mouse model of idelalisib resistance, resistant CLL cells exhibited increased mitogen-actived protein kinase (MAPK) signalling through enhanced expression of insulin-like growth factor 1 receptor (IGF1R), which could be abrogated using a small molecule inhibitor, lisitinib. Increased IGF1R expression was identified on CLL cells from one patient with progression on idelalisib, with restoration of idelalisib sensitivity when treated with lisitinib *in vitro*^[210]^. Whole exome sequencing experiments in this mouse model also identified frequent mutations relevant to integrin and extracellular matrix signalling^[211]^. Although promising, these preclinical observations are yet to be recapitulated in a larger analyses of patient samples.

Duvelisib (IPI-145) is a dual inhibitor of the d and g isoforms of PI3K^[212]^, postulated to impair the function of tumour-supporting CD4+ T cells and macrophages within the microenvironment through concomitant g isoform inhibition^[213-215]^. *In vitro* duvelisib interrupts AKT signalling within CLL cells stimulated by CD40L/IL-2/IL-10, CLL cell homing and intracellular signalling downstream of the BCR^[216]^. In a Phase I clinical trial, duvelisib attained an ORR of 56% (1 CR) in patients with R/R CLL and 83% in TN CLL, but was complicated by frequent toxicity (Grade ≥ III in 84% of patients), including diarrhea and transaminitis^[212]^. In a Phase III trial comparing duvelisib to ofatumumab in R/R CLL, the dual PI3K inhibitor showed superior ORR (74% *vs*. 45%) and PFS (13 months *vs*. 10 months), but a high rate of severe adverse effects (Grade ≥ III in 87% of patients: neutropenia 30%, diarrhea 15%, pneumonia 14% and anaemia 13%). Toxicity was the most common reason for discontinuing duvelisib (35% of cessations *vs*. 22% due to progressive disease)^[217]^. Overall, the modest PFS benefit and frequent toxicity that compromises idelalisib seems likely to similarly challenge the potential of duvelisib. Umbralisib (TGR-1202) is another PI3K inhibitor with greater selectivity for the p100d isoform as well as action against casein kinase-1e. In a Phase I study enrolling 90 patients with R/R CLL and non-Hodgkin lymphoma, serious adverse effects were largely haematological, although one case of severe colitis occurred, and 37% of patients had an objective response^[218]^. Preliminary data suggest that umbralisib may have a favourable toxicity profile and may be safely co-administered with ibrutinib or venetoclax with or without an anti-CD20 monoclonal; however, the follow up for these cohorts is short and serous immunological adverse events have occurred^[219-221]^. Longer-term follow up of alternative PI3K inhibitors will be required to determine whether they can indeed overcome the disadvantageous toxicities that vex idelalisib deliverability.

Another strategy to enhance the efficacy of BCR inhibitors has been to combine them with CIT regimens. In a Phase III study comparing idelalisib to placebo in combination with bendamustine-rituximab for R/R CLL, idelalisib combination was associated with prolonged PFS (median PFS 21 months *vs*. 11 months); however, it was associated with an increased rate of serious adverse effects (68% *vs*. 44%) and early discontinuation due to adverse event (27% *vs*. 13%)^[222]^. Although limited by cross study comparison, the PFS achieved by idelalisib-bendamustine-rituximab is similar to reports of idelalisib combined with rituximab or ofatumumab in patients with R/R CLL^[16,194,195,203]^. In a Phase I study, longer PFS was observed in patients receiving idelalisib-rituximab than idelalisib-bendamustine ± rituximab, although numbers were small^[202]^. Overall, these data cast doubt over the utility of combining idelalisib with chemotherapy in the R/R setting. Similarly, the HELIOS trial compared ibrutinib to placebo in combination with bendamustine-rituximab in patients with R/R CLL, with ibrutinib combination demonstrating superior depth of response, PFS (36-month PFS 68% *vs*. 14%) and OS (36 months OS 82% *vs*. 73%); however, Grade III/IV treatment emergent adverse effects (TEAEs) occurred in 89% of patients treated with triplet combination, with death due to TEAEs in 10% of patients^[149,223]^. Moreover, the 36-month PFS and OS observed with ibrutinib monotherapy in the RESONATE trial was 59% and 74%, respectively, suggesting that the yield of adding bendamustine may be low^[96]^. Although cross-trial comparison is unreliable, it remains unclear whether the addition of chemotherapy to novel agents in the R/R setting offers prolonged survival compared to novel agents alone or combined with anti-CD20 monoclonal antibodies. In the frontline setting, duvelisib has been combined with FCR for treatment of young, fit patients in a Phase Ib study, achieving BM uMRD status in 21 out of 26 (81%) patients, although severe immunological toxicities were observed^[224]^. Early data from a comparable Phase II trial adding ibrutinib to FCR in fit young patients with untreated CLL also demonstrated a high rate of BM uMRD attainment (84%)^[225]^, likely improved on historical outcomes with FCR alone^[226]^. One patient suffered from sudden cardiac death at 17 months, potentially attributable to ibrutinib^[225]^. Although these data suggest idelalisib or ibrutinib can enhance the depth of response to CIT, longer-term follow up and randomised studies will be required to determine whether the addition of chemotherapy, with likely enhanced toxicity, offers improved outcomes over targeted agents alone or in combination with each other.

### Summary: idelalisib

Although a valuable component of the therapeutic armamentarium against CLL, inferior disease control and frequent toxicity have limited the role of idelalisib in clinical practice. We would recommend venetoclax- or ibrutinib-based therapy over idelalisib for most patients with CLL, reserving idelalisib-rituximab therapy for patients who have disease resistant to both agents. Later generation PI3K inhibitors may have a role within fixed duration targeted therapy combination.

## Conclusions and future directions

Targeted agents have dramatically changed the therapeutic options and prognosis of patients with CLL. Most patients with CLL today are best treated with a targeted agent, with particular benefit in patients with traditional high-risk disease features or comorbidities that previously precluded effective therapy. Longer-term follow up may ultimately resolve the question of best first therapy in young fit patients with mutated IGHV. Older patients with mutated IGVH also have a long PFS and time to next therapy with chlorambucil-obinutuzumab and this may remain a reasonable or even preferred approach in settings where the cumulative community and personal cost of very-long-term ibrutinib is prohibitive, or cardiac toxicities are a concern. Unfortunately, another substantial pitfall of all three approved targeted agents is their significant cost, with an estimated monthly price of ~11,000 USD for ibrutinib, ~9000 USD for venetoclax and ~8000 USD for idelalisib^[227]^. The escalating costs of continuous targeted agent therapy threaten to stress societal and patient finances, but may be mitigated with the use of time-limited combination regimens. With maturing experience using continuous single agent targeted therapy, several common themes emerge. It is clear that indefinite BCR pathway inhibitor therapy is ultimately poorly tolerated by many patients, although alternative agents with different selectivity may improve deliverability. Nevertheless, even for patients who can tolerate long-term therapy with targeted agents, the emergence of resistant disease is common, at least in the R/R setting. For both venetoclax and ibrutinib, the presence of mutations in *TP53* or *NOTCH1*, and complex karyotype, portend the development of earlier resistant disease, despite high initial response rates. *BTK/PLCG2* mutations are present in the majority of patients relapsing on ibrutinib, whereas *BCL2* mutations account for only a portion of venetoclax resistance, with as yet unknown, but likely polyclonal, mechanisms driving progression in any individual patient. Pre-clinical evidence implicating the CLL microenvironment in venetoclax resistance provides a compelling case for ibrutinib-venetoclax combination. Indeed, it appears that ibrutinib and venetoclax are each an effective therapy for disease resistant to the other. Together, toxicities, inevitable resistance, the escalating cost of indefinite therapy and likely anti-neoplastic synergy form a robust argument for time-limited combination therapy, although long-term follow up will be required to evaluate this strategy. To date, the MURANO and CLL14 trials represent the most established steps toward time-limited combination, achieving deep remissions for the majority of patients (PB uMRD in 62% and 76%, respectively). Initial data from ibrutinib-venetoclax combination in high risk or elderly TN patients reported an ORR/CRR of 100%/88% after 12 cycles of combination therapy^[69]^, with 23 out of 29 (79%) patients who completed 24 months of combination therapy achieving BM uMRD status^[230]^. Similar rates of BM uMRD achievement (73%) have been reported after 12 cycles of ibrutinib-venetoclax treatment in younger TN patients in the CAPTIVATE study^[228]^. In patients with R/R CLL in the CLARITY study, at 12 months of ibrutinib-venetoclax combination, the ORR/CRR was 89%/51% with PB/BM uMRD attainment in 53%/36% of patients^[68]^. A Phase I trial of 12-month fixed duration venetoclax-ibrutinib-obinutuzumab in R/R patients reported an ORR/CRR of 92%/42% and 50% BM uMRD at the completion of treatment, allowing half the cohort to cease therapy while MRD-positive cases continued ibrutinib monotherapy. No new toxicities have yet been identified, although infusion reactions were common (83%)^[232]^. The same regimen in TN patients also appears safe and effective in an early report^[233]^. Combination of acalabrutinib-venetoclax-obinutuzumab has also demonstrated a favourable toxicity profile and 50% BM uMRD after eight cycles (four cycles of triplet therapy)^[229]^. Table 4 summarises clinical trials with a strategy for time-limited combination targeted agent therapy for which preliminary data are available. Overall, venetoclax is a critical component to facilitate time-limited therapy targeting uMRD, while planned use of continuous BTK inhibitor therapy for patients with suboptimal responses is a common theme among current trials. It seems certain that durable PFS and frequent uMRD remission will become commonplace with doublet or triplet targeted agent regimens. Extended follow up will be required to determine if any patients with CLL will achieve durable uMRD remissions with combination targeted therapy as seen with frontline FCR in IGHV mutated disease. As we migrate into a new paradigm of time-limited combination therapy, new questions emerge regarding the optimal combination, duration of therapy, predictors of safe cessation and possibility for re-treatment if the scourge of resistant disease is rendered infrequent.

**Table 4:** Targeted therapy combination trials with a strategy for treatment cessation

Study	Patient group	Fixed regimen	Cessation/continuation strategy	Preliminary data	Latest follow up
Tam *et al*.^[228]^ CAPTIVATE NCT02910583 (ibrutinib-venetoclax)	TN, < 70 years (*n* = 164)	IBR for cycle 1-3 IBR + VEN for cycle 4-15	BM uMRD or -pos after cycle 15 randomized to placebo or IBR*	After 12 combination cycles: PB uMRD 75% BM uMRD 72% 1 case laboratory TLS AEs leading to discontinuation in 7%	Median 15 (< 1-20) months
Lampson *et al*.^[229]^ NCT03580928 (acalabrutinib-venetoclax-obinutuzumab)	TN (*n* = 37)	ACAL for cycle 1-24 + G cycles 2-7 + VEN cycle 4-24	Patient may cease therapy at cycle 15 or cycle 24 if BM uMRD CR	After cycle 8 PB uMRD 65% BM uMRD 50% Grade ≥ III neutropenia 32% Infusion reactions 22%	Median 8 (2-11) months
Jain *et al*.^[69,230]^ NCT02756897 (ibrutinib-venetoclax)	TN Del(17p), mutated TP53, del(11q), unmutated IGHV or ≥ 65 years (*n* = 80)	IBR for cycle 1-3 IBR + VEN for cycle 4-27	BM MRD-pos patients after cycle 27 can continue IBR*	After cycle 27: 79% BM uMRD 12 patients (15%) off trial 3 cases laboratory TLS Grade ≥ III neutropenia 48%	Median 23 months
Jain *et al*.^[231]^ NCT02756897 (ibrutinib-venetoclax)	R/R (*n* = 80)	IBR for cycle 1-3 IBR + VEN for cycle 4-27	BM MRD-pos patients after cycle 27 can continue IBR*	After cycle 27: 67% BM uMRD 19% off trial, mostly due to toxicity	Median 22 months
Hillmen *et al*.^[68]^ CLARITY ISCRTN13751862 (ibrutinib-venetoclax)	R/R (*n* = 53)	IBR for 2 cycles IBR+VEN after cycle 2	PB/BM uMRD at cycle 8: cease therapy after cycle 14; PB/BM uMRD between cycle 14-26: cease therapy after cycle 26 MRD-pos at cycle 26: continue IBR monotherapy*	After 12 months combination: PB uMRD 53% BM uMRD 36% 34 episodes grade ≥ III neutropenia 1 case laboratory TLS	Median 21 months
Rogers *et al*.^[232]^ NCT02427451 (ibrutinib-venetoclax-obinutuzumab)	R/R (*n* = 12)	G cycle 1-8 + IBR cycles 2-14 + VEN cycle 3-14	After cycle 14, can continue IBR monotherapy*	After cycle 14: PB uMRD 100% BM uMRD 50% Grade ≥ III neutropenia 33% Infusion reaction 83%	Median 24 months
Rogers *et al*.^[233]^ (ibrutinib-venetoclax-obinutuzumab)	R/R (*n* = 25) TN (*n* = 25)	G cycle 1-8 + IBR cycles 2-14 + VEN cycle 3-14	After cycle 14, can continue IBR monotherapy*	Mid therapy, 16/23 (70%) BM uMRD Grade ≥ 3 neutropenia 56%, Grade ≥ 3 HTN in 32% 1 fatal neutropenic colitis	Median 18 (0-25) months
Niemann *et al*.^[234]^ VISION/HOVON 141 NCT03226301 (ibrutinib-venetoclax)	R/R (*n* = 230)	IBR for cycle 1-2 IBR + VEN for cycle 3-15	> PR and BM uMRD after cycle 15 randomized to observation or IBR monotherapy* MRD recrudescence retreated with combination	After 15 cycles: PB uMRD 55% BM uMRD 39% AEs leading to discontinuation in 4%	51 patients treated for duration of 15 cycles
Thompson *et al*.^[235]^ (ibrutinib-venetoclax)	≥ 1 year of prior IBR treatment, not uMRD High risk feature: TP53 mutation, del(17p), del(11q), CK, elevated B2MG (*n* = 35)	Add VEN to IBR therapy for up to 2 years	uMRD CR on two assessments: discontinue all therapy or continue IBR monotherapy* Patients MRD-pos or < CR: continue IBR monotherapy*	BM uMRD 10/15 (67%) at 12 months	15 patients evaluable at 12 months of combination therapy
Barr *et al*.^[219]^ NCT03801525 (umbralisib-venetoclax-ublituximab)	R/R (*n* = 21)	UMBRA cycle 1-12 + UBLI cycle 1-3 + VEN cycle 4-12	BM uMRD at cycle 12, discontinue all therapy BM MRD-pos at cycle 12, continue UMBRA monotherapy	4 patients with BM uMRD (19%)	Median 4 (< 1-14) months
Crombie *et al*.^[236]^ (duvelisib-venetoclax)	R/R (*n* = 12)	DUV D1-7, then DUV+VEN for 12 cycles	uMRD on two assessments, discontinue all therapy Resume VEN monotherapy at MRD recrudescence MRD-pos, continue VEN monotherapy	1 patient with BM uMRD CR Grade ≥ III neutropenia 83%	Median number of cycles 6 (range 1-9)

Cycles are 28 days unless otherwise stated. *Continued monotherapy until death. R/R: relapsed and refractory. TN: treatment naïve; R: rituximab; CIRS: cumulative illness rating scale; CrCl: creatinine clearance; PR: partial response; PB uMRD: peripheral blood measurable residual disease less than 10^-4^; BM uMRD: bone marrow measurable residual disease less than 10^-4^; IBR: ibrutinib 420 mg daily; ACAL: acalabrutinib 100 mg BD; VEN: venetoclax 400 mg daily after dose escalation; G: obinutuzumab (100 mg on Day 1, 900 mg on Day 2, 1000 mg on Days 8 and 15, then 1000 mg Day 1, 28 day cycles); UBLI: ublituximab 900 mg weekly for Cycle 1, then once for Cycles 2 and 3; UMBRA: umbralisib, 600 or 800 mg daily; DUV: duvelisib 25 mg BD; progression or unacceptable toxicity; CK: complex karyotype; B2MG: beta-2-microglobulin

## Declarations

### Copyright

© The Author(s) 2020.
